# Does the quality and outcomes framework reduce psychiatric admissions in people with serious mental illness? A regression analysis

**DOI:** 10.1136/bmjopen-2014-007342

**Published:** 2015-04-18

**Authors:** Nils Gutacker, Anne R Mason, Tony Kendrick, Maria Goddard, Hugh Gravelle, Simon Gilbody, Lauren Aylott, June Wainwright, Rowena Jacobs

**Affiliations:** 1 Centre for Health Economics, University of York, York, UK; 2 Primary Care and Population Sciences, University of Southampton, Aldermoor Health Centre, Southampton, UK; 3 Department of Health Sciences, University of York, York, UK; 4 Service User, UK

**Keywords:** MENTAL HEALTH, PRIMARY CARE, HEALTH ECONOMICS

## Abstract

**Background:**

The Quality and Outcomes Framework (QOF) incentivises general practices in England to provide proactive care for people with serious mental illness (SMI) including schizophrenia, bipolar disorder and other psychoses. Better proactive primary care may reduce the risk of psychiatric admissions to hospital, but this has never been tested empirically.

**Methods:**

The QOF data set included 8234 general practices in England from 2006/2007 to 2010/2011. Rates of hospital admissions with primary diagnoses of SMI or bipolar disorder were estimated from national routine hospital data and aggregated to practice level. Poisson regression was used to analyse associations.

**Results:**

Practices with higher achievement on the annual review for SMI patients (MH9), or that performed better on either of the two lithium indicators for bipolar patients (MH4 or MH5), had more psychiatric admissions. An additional 1% in achievement rates for MH9 was associated with an average increase in the annual practice admission rate of 0.19% (95% CI 0.10% to 0.28%) or 0.007 patients (95% CI 0.003 to 0.01).

**Conclusions:**

The positive association was contrary to expectation, but there are several possible explanations: better quality primary care may identify unmet need for secondary care; higher QOF achievement may not prevent the need for secondary care; individuals may receive their QOF checks postdischarge rather than prior to admission; individuals with more severe SMI may be more likely to be registered with practices with better QOF performance; and QOF may be a poor measure of the quality of care for people with SMI.

**Strengths and limitations of this study**

- This is the first study to investigate the relationship between general practitioner (GP) practice quality, as measured by four mental health Quality and Outcomes Framework (QOF) indicators, and psychiatric admissions in the English NHS.
- The data covered all practices in England and the results were found to be representative.
- The study used a consistent set of primary care quality indicators over the entire study period and employed longitudinal panel data estimation, therefore improving the robustness of results compared to previous research.
- A comprehensive set of GP practice and patient population characteristics were included in the models.
- An array of sensitivity analyses was undertaken and results were found to be robust.
- Aggregate practice-level data were used to examine the association between QOF and admissions and so we cannot be sure whether admitted patients had received a QOF review or had been exception reported.
- These are observational data and our results may be affected by unobserved confounders.

## Introduction

The quality of care of people with mental health problems is of international concern.^1^ ^2^ While primary care is central to the provision of mental healthcare in England, there is increasing focus on the interface between primary and secondary care, and the potential for better quality primary care to reduce avoidable hospital admissions and contain health expenditures.^3^ The role of general practitioners' (GP) care in preventing admissions is a matter of continuing debate,^4^ ^5^ including the mechanisms through which this might operate,^6^ but the tough economic climate implies this will be a subject of focused attention.^7^

Serious mental illness (SMI) includes schizophrenia, bipolar disorder and psychoses with considerable disability, prevalence^8–10^ and an estimated economic burden of £14 billion.^11^ In the UK, around 30% of people with SMI are treated solely by primary care clinicians^12^ and compared with those without mental health problems, people with SMI are in contact with primary care services for a longer cumulative time.^13^ ^14^ The pay-for-performance scheme in primary care—the Quality and Outcomes Framework (QOF)—includes targets to incentivise GPs to improve the quality of care for people with SMI.^15^

Although not an explicit aim of the QOF, several studies have examined whether better quality primary care can reduce hospital admissions. Analyses of relationships between QOF performance for coronary heart disease, asthma and chronic obstructive pulmonary disorder and hospital admissions found no effects,^16–18^ but better performance for diabetes^19^ ^20^ and stroke^21^ had a small negative association with emergency admissions. We hypothesise that this may be plausible for SMI. No previous study has tested this association for people with SMI. We investigated whether higher achievement on the SMI QOF indicators was associated with fewer psychiatric admissions for people with SMI.

## Methods

We carried out a retrospective analysis of routine data at GP practice level, estimating the effect of four QOF indicators (table 1) on psychiatric admissions to hospital using random effects Poisson regression.

**Table 1:** Overview of QOF indicators for SMI used in the analyses

Indicator	Description	Rationale
Care plan indicator [MH6]	The percentage of patients on the register who have a comprehensive care plan documented in the records agreed between individuals, their family and/or carers as appropriate	Reflects good professional practice and is supported by national clinical guidelines. A care plan should be accurate, easily understood, reviewed as part of the annual review and discussed with the patient, their family and/or carers. It should cover: Current health status and social care needs, including how needs are to be met, by whom, and the patient's expectations How socially supported the individual is, eg, friendships/family contacts/voluntary sector organisation involvement Coordination arrangements with secondary care and/or mental health services and a summary of what services are actually being received Occupational status Early warning signs (relapse signature) The patient's preferred course of action (discussed when well) in the event of a clinical relapse, including who to contact and wishes around medication
Review indicator [MH9]	The percentage of patients with schizophrenia, bipolar affective disorder and other psychoses with a review recorded in the preceding 15 months. In the review there should be evidence that the patient has been offered routine health promotion and prevention advice appropriate to their age, gender and health status	Patients with serious mental health problems are at considerably higher risk of physical ill-health than the general population, but are less likely to be offered health promotion advice. The annual review should cover: Accuracy of prescribed medication Issues related to alcohol/drug use Smoking and blood pressure Cholesterol checks BMI Risk of diabetes from olanzapine and risperidone An enquiry about cough, sputum, and wheeze^47^
Lithium indicator 1 [MH4]	The percentage of patients on lithium therapy with a record of serum creatinine and TSH in the preceding 15 months	Lithium monitoring is essential due to the narrow therapeutic range (0.6–1.0 mmol/L) of serum lithium and the potential toxicity from intercurrent illness, declining renal function or co-prescription of drugs, eg, thiazide diuretics or NSAIDs which may reduce lithium excretion. It is therefore necessary to check calcium and thyroid function on a regular basis as well as renal function. There is a much higher than normal incidence of hypercalcaemia and hypothyroidism in patients on lithium, and of abnormal renal function tests. Overt hypothyroidism has been found in between 8% and 15% of people on lithium
Lithium indicator 2 [MH5]	The percentage of patients on lithium therapy with a record of lithium levels in the therapeutic range within the previous 6 months	

*Sources*: QOF guidance.^23^ ^47^ ^48^

BMI, body mass index; NSAID, non-steroidal anti-inflammatory drug; QOF, Quality and Outcomes Framework; SMI, serious mental illness; TSH, thyroid-stimulating hormone.

### Sample

Our data set included all GP practices in the English NHS between April 2006 and March 2011. We chose this period because the QOF definition of SMI was constant and there was a stable set of mental health indicators. We excluded practices with fewer than 1000 registered patients within a year as unrepresentative of the way in which primary care is normally organised. Practices were also excluded if the registered number of patients with SMI was below 5 as their QOF achievement was prone to large variations over time. However, we did not apply this exclusion to the analysis of bipolar disorder because registered numbers of patients with bipolar disorder were low for most practices. We excluded practices reporting inconsistent numbers of patients with SMI or bipolar disorder across indicators within a year and practices where the registered number was fewer than the number admitted to hospital. Finally, we excluded all admissions for patients who changed practice within a year as it was unclear which practice affected the need for inpatient care.

### Data sources

We linked administrative data sets including the General and Personal Medical Statistics (GMS) data, the Attribution Data Set (ADS), the QOF data set and the annual GP Patient Survey data (table 2) using unique practice-year identifiers. Census (2001) data from the Office for National Statistics, measured at small-area level (ie, Lower Super Output Areas), were linked to practices on the basis of their practice population distribution as reported annually in ADS. Annual admission rates were calculated from Hospital Episodes Statistics (HES). All data sources are reported in the online supplementary appendix table 1.

**Table 2:** Covariates included in the regression models: descriptive statistics

Variable description	Data	Bipolar disorder				SMI			
	Source	Mean	SD	Minimum	Maximum	Mean	SD	Minimum	Maximum
GP practice characteristics									
=1 if GP practice is reimbursed under PMS	GMS	0.43	0.50	0	1	0.43	0.50	0	1
Proportion of male GPs/practice	GMS	0.60	0.27	0	1	0.61	0.27	0	1
Proportion of non-UK qualified GPs/practice	GMS	0.31	0.36	0	1	0.33	0.38	0	1
Mean age of GPs/practice	GMS	47.68	7.35	28	76	48.05	7.65	28	76
Practice list size	ADS	6899	4008	1040	40 082	6707	4008	1040	40 082
Characteristics of the practice population									
Patient population: average age	ADS	39.08	4.05	21.97	56.43	38.91	4.15	21.56	56.43
Patient population: proportion of male patients	ADS	0.50	0.02	0.38	0.78	0.50	0.02	0.38	0.79
Proportion claiming incapacity benefit for mental health, practice catchment area	DWP	0.02	0.01	0.00	0.07	0.02	0.01	0.00	0.07
Proportion providing informal care, practice catchment area	ONS	0.10	0.01	0.05	0.15	0.10	0.01	0.05	0.15
NHS psychiatric residents per 1000 population, practice catchment area	ONS	0.19	1.10	0	63.57	0.19	1.12	0	63.57
Proportion of non-white ethnicity, practice catchment area	ONS	0.11	0.15	0.00	0.80	0.11	0.16	0.00	0.81
Proportion living in urban setting, practice catchment area	ONS	0.82	0.33	0	1	0.82	0.33	0	1
Measures of access to care									
Distance (in miles) from practice to closest acute hospital	HES	4.85	4.98	0	59.44	4.74	4.91	0	59.44
Distance (in miles) from practice to closest mental health hospital	HES	10.71	8.35	0	74.05	10.55	8.27	0	74.05
Proportion of practice patients able to access care within 48 h	GPPS	0.84	0.11	0	1	0.84	0.11	0	1
Baseline admissions									
Mean number of admissions between April 2004 and March 2006	HES	1.58	1.77	0	42	4.37	4.29	0	63

ADS, Attribution Data Set; DWP, Department for Work and Pensions; GMS, General Medical Services; GP, general practitioner; GPPS, GP patient survey; HES, Hospital Episode Statistics; ONS, Office for National Statistics, Neighbourhood Statistics; PMS, Personal Medical Services; SMI; serious mental illness.

### Hospital admissions

HES records diagnoses using the International Classification of Diseases (ICD) 10 classification system, whereas the QOF inclusion criteria are based on Read codes used in primary care. To identify the relevant population, we used the Health and Social Care Information Centre cross-mapping from Read to ICD-10. Psychiatric admissions were defined as hospital inpatient episodes in patients aged 18 years or over with a main diagnosis of SMI (ICD-10 codes: F20-F31); for the subset of bipolar disorder admissions, defining main diagnoses were ICD-10 codes F30-F31.

Just under one-quarter (23.3%) of psychiatric admissions in our data were coded as elective. However, some providers class all mental health admissions as emergencies; hence, elective and emergency psychiatric admissions cannot be consistently distinguished in routine data due to variation in coding.^22^ On the advice of our study steering group (including policy experts, clinicians and people with SMI) we therefore pooled all admissions irrespective of how they were coded by the provider, and conducted sensitivity analyses.

### Measures of practice quality

During our study period, the QOF mental health domain included five indicators to incentivise proactive disease management for a population where low adherence to medication, or drug levels outside of a therapeutic range, may lead to relapse and hospitalisation (table 1).^23^ Two of these indicators (MH6 and MH9) apply to all registered patients with a diagnosis of SMI, whereas the two lithium indicators (MH4 and MH5) apply only to patients with bipolar disorder. Our analyses excluded indicator MH7, which encourages follow-up of patients with SMI failing to attend their annual review, because practices that review all eligible patients cannot score on MH7 and so would not contribute to the analysis. We selected indicators on the basis of consistency over time within our study period (see online supplementary appendix table 2).

Under the QOF, practices may ‘exception report’ patients,^24^ that is, remove inappropriate patients from the denominator used to calculate achievement. While exception reporting may reflect good-quality care, for example, because patients are deemed unsuitable for clinical reasons, it could also reflect ‘gaming’ by GPs, who can increase the number of points they earn by reducing the eligible population inappropriately.^25^

The set of patients for each QOF indicator (all patients with SMI for MH6 and MH9, or all patients with bipolar disorder for MH4 and MH5) were divided into three mutually exclusive categories: those for whom the indicator was achieved (A), those who were exception reported (E), and those for whom the indicator was not achieved (NA). For each indicator, we calculated two measures of practice performance. The QOF incentive regime rewards GPs on the basis of reported achievement:1
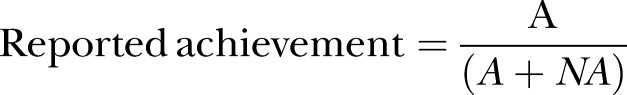
which is set to 0 if all patients are exception reported.

Given that the appropriate level of exception reporting is uncertain and we cannot distinguish admissions for patients who were exception reported from those who were not, we followed Kontopantelis *et al*^26^ in using:2
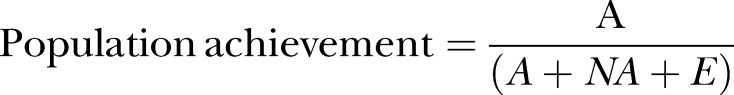
as our preferred practice performance measure in our main analysis. We used three sensitivity analyses to investigate the effect of including exceptions in calculating achievement. First, proportions of exception-reported patients included in the denominator were successively increased by 10 percentage points from 0% (equation 1) to 100% (equation 2) to identify potential switching points (ie, the levels at which the sign and statistical significance of the estimated coefficient changed). Second, we stratified practices into tercile groups by their level of exception reporting and interacted the respective achievement rate with the population exception rate:3
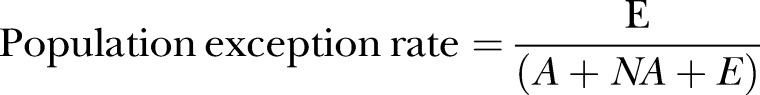
This sensitivity analysis was restricted to indicators MH6 and MH9 as there was insufficient variation in exception rates for the bipolar indicators to classify them into terciles. Finally, we also tested a model including the population exception rate as a separate covariate.

### Covariates

Data on hospital admissions and practice quality were linked to GP practice characteristics, their patient population characteristics and population characteristics including deprivation and other potential confounders recorded at small-area level (table 2). We also controlled for measures of access to care and modelled Primary Care Trust fixed effects to account for differences in resourcing of crisis resolution and home treatment teams providing alternative home care in emergencies and playing a ‘gatekeeping’ role in hospital admissions.^27–29^ Year indicator variables were used to account for temporal trends. In order to reduce potential bias from unobserved practice-specific confounders, we included presample baseline admission numbers per practice (averaging financial years 2003/2004 to 2005/2006).^30^

### Analysis

Random effects Poisson regression models were estimated to relate the number of psychiatric admissions per practice to its QOF achievement, conditioning on potential confounding factors and a normally distributed GP practice random effect with zero mean and constant variance.^31^ ^32^ The numbers of practice-registered patients with SMI or bipolar disorder were used as exposure terms. We obtained cluster-robust Huber-White SEs to account for potential overdispersion.^31^ Coefficient estimates are presented as incidence rate ratios (IRR) with 95% CIs, so that a coefficient less than 1 indicates that the variable reduced admissions and vice versa. We also calculated the average effect of a 1% increase in QOF achievement on admissions. All analyses were conducted in Stata V.13.

Separate models were estimated for the SMI and bipolar admissions. The model for all SMI admissions included MH6 and MH9 as explanatory variables and for bipolar admissions included MH4 and MH5. Achievement scores were introduced as sets because in practice they are likely to be achieved jointly (the Pearson's correlation between MH4 and MH5 was 0.369, and between MH6 and MH9 was 0.585).

We conducted several additional robustness checks. First, we tested the effect of including QOF indicators separately (rather than as sets). Second, we estimated the model only on within-practice variation, where any time-constant (un)observed practice effects were conditioned out of the likelihood. Third, we used a dependent variable based on numbers of individuals admitted at least once in any given year (rather than total numbers of admissions) to test whether individuals admitted frequently (‘revolving door’ cases)^33^ distorted observed admission rates. Fourth, we tested the effect of using only admissions coded as emergencies. Fifth, we ran models using 1-year, 2-year or 3-year lags between QOF scores and admissions. Lastly, we estimated separate (cross-sectional) models for each study year to check for temporal effects not already accounted for in our regressions.

## Results

### Descriptive statistics

Our sample included 8234 GP practices that treated people with SMI during the 5-year period (38 774 practice-year observations; mean follow-up 4.8 years). The number of practices (8052) and practice-year observations (37 573) were lower for the bipolar sample because not all practices treating people with SMI also treated people in the subsample of bipolar disorder. The median number of people with SMI per practice was 39 (interquartile range (IQR)=22–64) and the median number of people with bipolar disorder was 6 (IQR=3–10). The median number of annual admissions per practice was 3.5 (IQR=1–5) for SMI, and 1.1 (IQR=0–2) for bipolar disorder.

Over time, average practice QOF achievement improved across all four indicators, whereas the exception-reporting rate declined (table 3).

**Table 3:** Average practice population Quality and Outcomes Framework (QOF) achievement and exception rates, 2006/2007 to 2010/2011

Financial year	Population achievement rate (%)				Exception reporting rate (%)			
	MH6	MH9	MH4	MH5	MH6	MH9	MH4	MH5
2006/2007	64.3	79.9	93.7	82.1	15.8	13.0	3.2	8.9
2007/2008	72.7	81.0	93.8	82.3	13.7	12.9	3.5	9.6
2008/2009	76.8	81.1	94.4	82.6	12.1	12.7	3.1	9.3
2009/2010	81.3	81.5	95.0	82.9	8.9	12.0	3.0	9.2
2010/2011	82.4	81.9	96.0	84.4	8.2	11.9	2.4	8.4
Pooled	75.5	81.1	94.6	82.9	11.7	12.5	3.0	9.1

### Main analysis

There was a consistent positive association between QOF achievement rates and hospital admissions for all indicators apart from MH6 (documented comprehensive care plan) (table 4). For MH9 (annual review), an additional 1% in achievement rates was associated with an average increase in the practice admission rate of 0.19% (95% CI 0.10% to 0.28%) or 0.007 patients (95% CI 0.003 to 0.01). Corresponding figures for bipolar disorder indicators were 0.16% (95% CI 0.01% to 0.30%) or 0.002 patients (95% CI 0.0001 to 0.003) for MH4 (thyroid-stimulating hormone (TSH)/creatinine checks for those on lithium) and 0.10% (95% CI 0.01% to 0.19%) or 0.001 patients (95% CI 0.0001 to 0.002) for MH5 (lithium level within therapeutic range).

**Table 4:** Regression results for main specification and sensitivity analyses based on population achievement rates

Model description	Admissions for bipolar disorder					Admissions for SMI				
		MH4		MH5			MH6		MH9	
	N	IRR	95% CI	IRR	95% CI	N	IRR	95% CI	IRR	95% CI
Main analysis	37 037	1.169	(1.011 to 1.353)	1.106	(1.012 to 1.209)	38 774	1.018	(0.941 to 1.100)	1.209	(1.104 to 1.324)
Sensitivity analyses										
Separate inclusion of indicators	37 037	1.235	(1.079 to 1.413)	1.141	(1.051 to 1.240)	38 774	1.110	(1.038 to 1.188)	1.223	(1.131 to 1.323)
Patient admitted at least once	37 037	1.108	(0.966 to 1.272)	1.116	(1.028 to 1.212)	38 774	1.012	(0.944 to 1.084)	1.254	(1.155 to 1.362)
Only emergency admissions	37 037	1.091	(0.936 to 1.271)	1.115	(1.014 to 1.226)	38 774	0.986	(0.908 to 1.071)	1.249	(1.132 to 1.379)
Achievement lagged by 1 year	29 030	1.095	(0.941 to 1.273)	0.958	(0.872 to 1.053)	30 604	0.998	(0.932 to 1.069)	1.184	(1.088 to 1.289)
Achievement lagged by 2 years	21 241	0.982	(0.819 to 1.178)	1.005	(0.899 to 1.124)	22 675	1.074	(0.994 to 1.161)	1.047	(0.948 to 1.155)
Achievement lagged by 3 years	13 863	0.865	(0.698 to 1.071)	0.940	(0.822 to 1.076)	15 059	1.005	(0.917 to 1.102)	1.219	(1.079 to 1.377)
Cross-sectional (year=2006)	7239	1.119	(0.825 to 1.519)	1.055	(0.856 to 1.299)	7605	1.096	(0.969 to 1.240)	1.342	(1.123 to 1.602)
Cross-sectional (year=2007)	7526	1.027	(0.758 to 1.392)	1.099	(0.913 to 1.323)	7880	0.989	(0.861 to 1.137)	1.342	(1.124 to 1.602)
Cross-sectional (year=2008)	7441	0.999	(0.720 to 1.386)	0.935	(0.768 to 1.139)	7750	0.990	(0.858 to 1.141)	1.230	(1.030 to 1.468)
Cross-sectional (year=2009)	7343	1.024	(0.694 to 1.510)	0.986	(0.802 to 1.214)	7691	1.096	(0.937 to 1.283)	1.024	(0.854 to 1.227)
Cross-sectional (year=2010)	7448	1.179	(0.803 to 1.731)	1.006	(0.816 to 1.240)	7848	0.956	(0.814 to 1.123)	1.193	(0.996 to 1.429)
Modelling exception rate separately	37 037	1.281	(1.043 to 1.574)	1.076	(0.959 to 1.207)	38 774	0.988	(0.907 to 1.076)	1.055	(0.931 to 1.195)
Interaction with exception rate: low (1st tercile)							1.014	(0.937 to 1.097)	1.166	(1.054 to 1.291)
Interaction with exception rate: medium (2nd tercile)							1.013	(0.934 to 1.098)	1.158	(1.041 to 1.287)
Interaction with exception rate: high (3rd tercile)							0.987	(0.902 to 1.081)	1.149	(1.018 to 1.297)
Within practice variation only (GP fixed effects)	32 328	1.241	(1.066 to 1.446)	1.209	(1.101 to 1.329)	37 818	1.048	(0.942 to 1.166)	1.163	(1.038 to 1.304)

All associations are expressed as IRRs.

IRR, incidence rate ratio; N, number of practice-year observations; SMI; serious mental illness.

**Figure 1:**
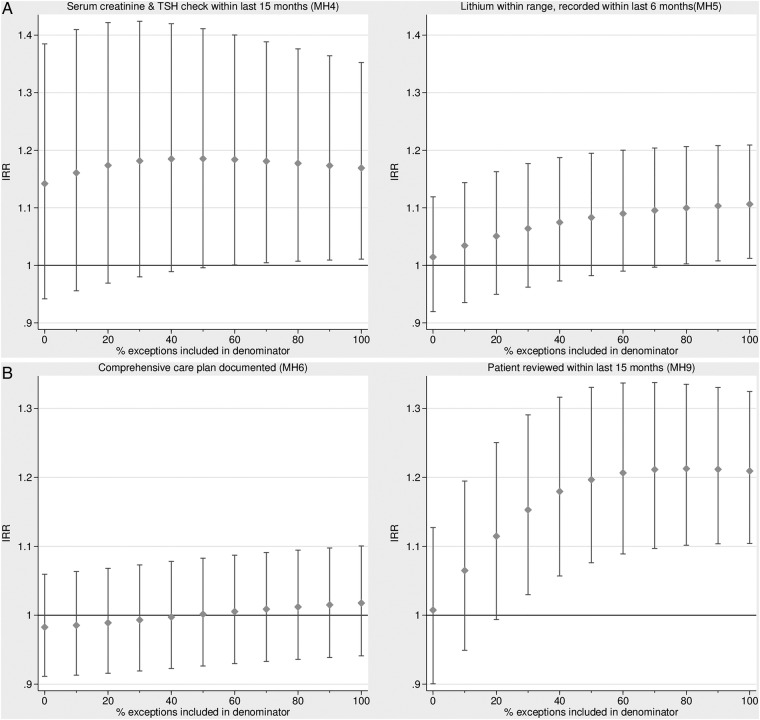
Association between achievement on the Quality and Outcomes Framework (QOF) indicators and admission rates (incidence rate ratios), by percentage of exception-reported patients included in the denominator. (A) Admissions for patients with serious mental illness (SMI). (B) Admissions for patients with bipolar disorder (IRR, incidence rate ratio; TSH, thyroid-stimulating hormone).

Reported achievement, where exception-reported patients were excluded, was not statistically significantly associated with admissions for any of the four indicators (see online supplementary appendix table 3). However, the association between achievement on MH9 and psychiatric admissions was significant when at least 30% of exception-reported patients were included in the denominator (figure 1). Given the median SMI practice register of 39 patients, the overall (pooled) MH9 exception rate of 12.5% (table 3), and a switching point of 30%, we calculated that approximately 1.5 exception-reported patients per practice year (=39×0.125×0.3) needed to be included in the denominator for the positive effect of MH9 on admissions to be statistically significant. The association between QOF achievement on MH4 [MH5] and admissions for bipolar disorder was statistically significant if at least 0.02 [0.08] exception-reported patients were included in the denominator.

Covariates generally had anticipated plausible and significant effects (see online supplementary appendix table 4 for results of all coefficients for the main specifications). Results for reported achievement rates (equation 1) for the main analyses and sensitivity checks are provided in online supplementary appendix table 3.

### Sensitivity checks

Findings were generally robust to sensitivity analyses, including patients admitted at least once, within-practice effects, lagged QOF achievement scores and for only admissions coded as emergencies (table 4) although results were not always statistically significant. The effect of population achievement rates on admissions was similar across practices with high/medium/low exception rates. When the model also included the separate population exception rate, only achievement on MH4 had a positive and statistically significant effect on admissions. For all indicators, population achievement was significantly associated with higher admissions if the indicators were included separately rather than together. Cross-sectional analyses broadly supported the results for MH9, but showed that the estimates’ association diminished over time. In addition, the lithium indicators (MH4 and MH5) were not significantly associated with admissions in individual years. None of the sensitivity checks found that the QOF indicators were associated with significantly lower hospital admissions.

## Discussion

Our study is the first to investigate the relationship between GP practice quality, as measured by four mental health QOF indicators, and psychiatric admissions in the English NHS. Contrary to expectations, we found that better performance was associated with a higher psychiatric admission rate on three of these indicators, although the likely magnitude of any effect was small.

The potential for higher quality primary care to reduce emergency admissions is an important issue^3^ ^7^ and existing research has addressed this in several disease areas, with mixed results.^34–37^ The evidence on the effectiveness of the QOF on admissions is similarly mixed.^16–21^ However, we are not aware of any finding that better quality care is associated with a significantly higher rate of admissions. Our study used longitudinal panel data rather than cross-sectional data, which allows us to control for unobserved time-constant confounders, therefore improving the robustness of results compared with previous research.

We explored the robustness of results to variations in exception reporting in practices and showed the results were sensitive to this. A previous study examining exception reporting found wide variation across practices and across indicators.^38^ We found a high level of exception reporting for MH9. These may be because a patient is deemed unsuitable for clinical reasons, or because a patient received at least three invitations for review during the preceding 12 months, but did not attend, or they refused to be treated. It is not possible to identify an appropriate level of exception reporting, but some people with SMI may be harder to reach due to the nature of their mental health problems; thus, practices may face difficulties in establishing and maintaining contact, and some degree of exception reporting is, therefore, expected.

There are a number of limitations to our study. As with all observational studies, we cannot ascertain causality. Our results may be affected by unobserved time-varying confounders that correlate with achievement rates. If these factors are positively associated with admission rates and with achievement rates then our estimate of the effect of achievement rate on admissions will be biased upwards. Also, since QOF data are reported at practice level, we cannot be sure whether admitted patients had been reviewed or exception reported. This makes it difficult to ascertain timing and causality. For example, it is possible that those admitted then received a QOF check arranged on discharge from hospital which could explain the positive association. Alternatively, patients with SMI whose problems are more severe may be preferentially registered with practices that are better equipped to provide their care. These practices may achieve higher QOF scores, but also uncover more unmet needs and have more admissions because of their case-mix. Both explanations would imply that the estimated positive association between QOF performance and admissions is not causal.

A second data limitation is that a few of our control variables are time invariant because they are based on census data. It may be possible that the underlying factors (eg, the ethnic composition of neighbourhoods) have changed over our study period, which may bias our results in unknown directions.

Two further limitations arise from our defined inclusion criteria and outcomes. First, we counted all admissions for people with bipolar disorder even though some may not have received lithium therapy, reducing the likelihood of finding effects for MH4 or MH5. Second, we implicitly assumed that psychiatric admission is a poor outcome. However, QOF checks may uncover mental health problems best addressed by admission and we cannot distinguish appropriate admissions from those representing avoidable failures in care.

Finally, it is possible that QOF indicators do not accurately measure the quality of primary care for SMI. The QOF, like any other pay-for-performance scheme, may result in tunnel vision^39^ or a focus on areas of activities which are incentivised, sometimes at the expense of other non-incentivised activities.^40^ Thus, high QOF attainment may not necessarily reflect high-quality care.

Further avenues are ripe for exploration including analysis at the patient level, rather than practice level, which would allow for detailed exploration of the entire patient pathway, including identification of the timing of QOF checks for admitted patients. Other priorities include consideration of non-QOF measures of primary care quality that might reduce admissions more effectively and could be incentivised through pay-for-performance; identification of types of secondary care for which utilisation may be affected more by primary care; and investigating whether some practices are more successful than others in getting patients admitted and whether this correlates with their QOF achievement, particularly relevant in the light of high occupancy levels due to closures of mental health beds.^41^ Finally, greater understanding of unmet needs for people with SMI is essential.^42^ Prevalence of an unmet need relates to mental healthcare provision and to socioeconomic circumstances—the less integrated and continuous care and the poorer the life situation, the higher is the unmet need.^43^ More precise estimates of unmet needs can inform policy initiatives to ensure primary care is appropriately equipped and incentivised.

While current policy places an emphasis on ‘upstream’ prevention and ‘early intervention’ to reduce the need for more intensive and expensive specialist care,^44^ ^45^ our findings raise doubt about whether improvements in primary care quality, as measured by the QOF, are likely to achieve this. This chimes more broadly with concerns about the effectiveness of current approaches to reduce avoidable secondary care use.^46^ While the QOF was not explicitly designed to reduce hospital admissions, there may be effective alternative primary care interventions that could be incentivised in the QOF.
